# Virtual Labs: E-Learning for Tomorrow

**DOI:** 10.1371/journal.pbio.0020157

**Published:** 2004-06-15

**Authors:** Camillan Huang

## Abstract

At Stanford University, the Virtual Labs project takes full advantage of information technology to provide innovative resources for learning and teaching

Because of the explosive growth in our scientific understanding, today's students are required to learn and maintain a rapidly expanding knowledge base. Students are also expected to understand and follow the crossover of information between different disciplines. As a result, they often have to understand the fundamentals of several disciplines, and be able to integrate that knowledge.

Students of every discipline are facing these new challenges, and it is clear that today's students are markedly different from those of the past. Influenced by a lifetime surrounded by media, computers, and the Internet, they bring with them different expectations. As educators, we need to meet these expectations in order to motivate students to move forward.

And it's not just the student population that is driving change. The National Institutes of Health, which sponsors many biological and medical advances in the United States, has a new initiative called “Digital Biology: The Emerging Paradigm,” whose goal is to merge biomedical computation with biology and medicine over the next ten years. One way to facilitate this movement is to use information technology (IT) as a teaching tool, so that students, in turn, learn how to use IT most effectively.

## Using IT to Teach

IT presents educators and teachers with a unique opportunity to devise innovative methods of teaching. Students today are more likely than ever to use new tools and technologies to advance their understanding of the sciences. Currently, this usage is mainly limited to searching the Web for information. However, computers and the Web can be used for much more—with computers, you can create learning scenarios like virtual patient simulations, and with the Web, these learning resources can be disseminated to the global community. Educators must harness the power of these enabling technologies, which students have already adopted, to create new and more powerful methods of teaching that will better prepare the students for the next phase of their lives.

The Virtual Labs Project at SUMMIT (Stanford University Medical Media and Information Technologies) in the Stanford University School of Medicine has been funded by the Howard Hughes Medical Institute (Chevy Chase, Maryland, United States) since 1998. It is an initiative to augment the Department of Biological Sciences and the Program in Human Biology at Stanford University by developing technology-enhanced materials for these curricula. A major goal of the Virtual Labs Project is to increase scientific literacy by using interactive multimedia to teach the fundamental concepts of biology, and to share those resources via the Internet. The Virtual Labs material is currently hosted on a password-protected site and is freely available to interested parties for educational use.

A wise individual once said that a picture is worth a thousand words; with Virtual Labs we use not just pictures, but also animations and interactive simulations. Students are able to visualize and interact with dynamic processes in the body. We have developed learning modules in cardiovascular, gastrointestinal, respiratory, renal (Video 1), visual (Video 2), and neurophysiological systems. The concepts in these modules lay the foundation for medicine and for an increasing number of interdisciplinary programs, such as biomedicine, medical informatics, and bioengineering. For example, a medical student learns how the kidney filters blood in order to understand kidney failure in diabetic patients. A bioengineer could apply the same knowledge to build an artificial kidney. The modules are flexible, and the content can be woven together to highlight the intersections of different disciplines.

Virtual Labs also strives to make learning science fun. The more engaged the user is, the more likely the learning experience is to be positive. For example, after learning about how the kidney filters blood (see the online link for Figure 1) and controls water levels, students apply their new knowledge by playing a simulation game. The goal of the game is to maintain water balance in order to survive on a deserted island, which helps to reinforce conceptual understanding and to ensure that students understand how those concepts fit together.

Responses from students have shown that these goals are being met. Over the past four years, our undergraduate and medical students have reported that Virtual Labs was fun, engaging, and that it helped them learn: “Virtual Labs was an excellent resource for the class! [It was] a lot of fun to use and the graphics are awesome.” “It was a great interactive way to reinforce what I already had learned from the book and lectures and I think it really helped me better understand.” Similarly, faculty who have used our material during lectures have found it useful to illustrate concepts with animations.

## Reaching Beyond the Local Community

Local schools and other universities are looking for opportunities to bring IT into their classrooms. Access to resources like Virtual Labs, and expertise on how to develop and integrate multimedia content into curricula, are on the rise. In 2003, the Virtual Labs Project began building a network of collaborators in the community and abroad. Together with H.E.L.P for Kids (http://www.stanford.edu/group/help,) we are designing content for the education of local schoolchildren. Abroad, we are working with global partners in Sweden via the Wallenberg Global Learning Network (http://www.wgln.org). We have also partnered with the MedFarmDoIT group at Uppsala University in Uppsala, Sweden (http://doit.medfarm.uu.se/multimedia.html) to help them with multimedia development and IT integration in the classroom. The MedFarmDoIT group shares our vision of bringing IT into the classroom, and together, we are designing content for their medicine/pharmacy program.

The Virtual Labs Project is dedicated to supporting its partners by distributing customized Virtual Labs content and offering consultation or workshops to train teachers and developers. As integrated partners, we can bridge the gap between the physical and information sciences, and in doing so, can improve the learning process of students for years to come.

## 

**Video 1:** The “Big Picture” of the Blood Flow through the Vasculature in the Kidney The rich visuals and moving media of this Virtual Labs animation capture the attention of the students. (The animation can be accessed online on computers with Shockwave by clicking and dragging the file into the browser window. A free version of Shockwave can be downloaded from http://sdc.shockwave.com/shockwave/download.)

**Video 2:** Understanding Center–Surround Receptive Fields in Retinal Neurons The virtual experiment in this Virtual Labs interactive program is similar in design to the receptive field experiments from Hubel and Wiesel in the 1960s. The user places an electrode in the retina to take a recording from a neuron. The user moves a spot of light on the screen and then maps correlating changes in the activity level (using the symbols: + − 0 to indicate the strength of the response). The map reveals a center–surround organization. Each time the user moves the electrode, the size, shape, location, and type of receptive field changes (on-center or off-center), as they would during a real experiment. Supporting questions adapt dynamically to each experimental condition and further encourage the student to answer more conceptual questions. (The interactive program can be accessed online on computers with Shockwave by clicking and dragging the file into the browser window. A free version of Shockwave can be downloaded from http://sdc.shockwave.com/shockwave/download.)

## Abbreviations

IT: information technology
SUMMIT: Stanford University Medical Media and Information Technologies
